# Multi-year belowground data of minirhizotron facilities in Selhausen

**DOI:** 10.1038/s41597-023-02570-9

**Published:** 2023-10-03

**Authors:** Lena Lärm, Felix Maximilian Bauer, Normen Hermes, Jan van der Kruk, Harry Vereecken, Jan Vanderborght, Thuy Huu Nguyen, Gina Lopez, Sabine Julia Seidel, Frank Ewert, Andrea Schnepf, Anja Klotzsche

**Affiliations:** 1 Institute of Bio-and Geosciences, Agrosphere (IBG-3), Forschungszentrum Jülich GmbH, Jülich, 52425 Germany; 2 Crop Science Group, Institute of Crop Science and Resource Conservation (INRES), University of Bonn, Bonn, 53115 Germany; 3 Leibniz Centre for Agricultural Landscape Research (ZALF), Müncheberg, 15374 Germany

**Keywords:** Environmental sciences, Plant sciences, Hydrology

## Abstract

The production of crops secure the human food supply, but climate change is bringing new challenges. Dynamic plant growth and corresponding environmental data are required to uncover phenotypic crop responses to the changing environment. There are many datasets on above-ground organs of crops, but roots and the surrounding soil are rarely the subject of longer term studies. Here, we present what we believe to be the first comprehensive collection of root and soil data, obtained at two minirhizotron facilities located close together that have the same local climate but differ in soil type. Both facilities have 7m-long horizontal tubes at several depths that were used for crosshole ground-penetrating radar and minirhizotron camera systems. Soil sensors provide observations at a high temporal and spatial resolution. The ongoing measurements cover five years of maize and wheat trials, including drought stress treatments and crop mixtures. We make the processed data available for use in investigating the processes within the soil–plant continuum and the root images to develop and compare image analysis methods.

## Background & Summary

As a result of climate change, ensuring food security for the vastly growing human population is one of the major challenges of the 21st century. While climate change is exerting increasing pressure on the availability of natural resources such as water and soil nutrients, there is an increasing demand on food production. To ensure food security for the growing world population, agricultural production will have to increase by at least 60% by 2050^1^. The yield of agricultural crops therefore needs to be increased and yield stability under changing conditions must be preserved, if current consumption patterns are maintained. A comprehensive understanding of all processes within agro-ecosystems is crucial to identify the key parameters to maintain yield stability and increase yield. The main source of water and nutrients for plants is the rhizosphere and the surrounding soil. Key parameters for potential improvements in water and nutrient efficiency could be revealed through a comprehensive understanding of the soil–plant continuum and its processes. This includes parameters describing the root architecture, influencing processes such as root water, and nutrient uptake, which governs the yield^2^. Field phenotyping, especially incorporating below ground information is crucial for breeders to capitalize on developments in genetics, since information identified under controlled environment are often not accounting for “real-world“ field conditions^3^. In-field observations also enable to investigate quantitative traits, particularly those related to root features that influence drought stress tolerance. Therefore, field phenotyping facilities including below ground information provide precious data for breeders^4^. Additionally, knowledge about soil heterogeneity is crucial to understanding the distribution in soil water and nutrient content.

The data presented here include information about crop-relevant subsoil data – such as soil water content, soil water potential, soil temperature, and root development – on a high temporal-spatial resolution for multiple crop growing periods.

There are several techniques to observe roots non-destructive. The whole root system development can be observed with rhizotrons, equipped with a clear window on the side. Rhizotrons exist in various shapes for greenhouse and in-field observation^5,6^. If installed above ground, these rhizoboxes allow for the sampling and imaging of root systems through easily accessible windows and apertures at the side^7,8^. In the past, several in-field rhizotrons often took the form of covered underground cellars or walkways with transparent windows or side walls for observing root development. In order to avoid expensive construction and maintenance costs, transparent – minirhizotrons (MR) – were introduced, enabling the *in situ* observation of the root in a fixed position, but at several depths^9^. By installing transparent tubes with an inclination, they could be accessed from the surface. These rhizotubes were subsequently also used in rhizotron facilities, where they were installed horizontally from the trench walls at different depths to ensure that root distributions and root development could be observed in a larger soil volume than only at the side walls^10^. It is important that the installation of the rhizotubes is causing as little soil disturbance as possible. Especially in fine textured soil, less soil compaction around the tube, caused by the installation process, might alter the root growth^11^. These influences on the collected root data can be reduced to a negligible minimum when auger with the same diameter as the rhizotubes are used to drill holes for tube insertion, the soil is re-compacted according to previous bulk density measurements and a resting period is respected after tube installation (6–17 month)^11–14^. The permanent installation and maintenance of MR at several depths has only been done on very rare occasions due to the high manufacturing effort involved^10,15^. However, this kind of MR facility enables insights into processes within the soil–plant continuum at the plot scale, while offering high instrumentation for multifaceted observations at high spatial and temporal resolution.

One way to observe the root growth is imaging the roots and surrounding soil through the transparent rhizotubes with a special camera system. To analyze the resulting root images, various methods from root counting to single root analysis were performed with several manual or semi-automated software tools^14,16–18^. Depending on the targeted phenotypic traits and root image quality it is not always feasible to extract it manually from the images^14,19^. In contrast to genotype analysis, which can be performed with various high-throughput methods, the phenotyping of corresponding plant architecture and anatomy is still a bottleneck^20^. Image analysis based on the convolutional neural network (CNN) is the most promising way to close this gap^21^. In particular, CNNs are used to automatically detect different plant organs by segmenting them from the background^22^. While this is already established for above-soil organs of plants, applying these techniques to extract information about the root system remains challenging, especially under field conditions^23,24^. This is mainly due to the lack of availability of root image data, which are required to train a segmentation model, compared to shoot image data. Capturing shoot images is inexpensive and easy, while in-field root imaging is time- and labor-intensive (image acquisition time is 5–10 minutes on average per tube)^19,25^.

In addition to the root information, soil sensors measure point information on soil water content, soil water potential and soil temperature. Moreover, the spatial soil water content per depth can be measured with a ground-penetrating radar (GPR)^26,27^ between two neighboring rhizotubes.

The two MR facilities^28^ in Selhausen, Germany, enable longer term studies of the soil–plant continuum on two different soils in the same climate. To investigate the different components of the soil–plant continuum, these MR facilities offer unique conditions to record 4D subsoil information for multiple growing seasons under different field conditions and agronomic treatments. Detailed information about soil water content (SWC), soil water potential, and soil temperature was obtained at two locations within different soil types by the soil sensors mentioned above. Furthermore, morphological root information was obtained *in situ*, including relevant root system traits such as length, diameter, branching frequency, etc. Root traits were acquired with cameras, taking images through horizontal transparent rhizotubes installed at several depths^28,29^. Since all measures to avoid altered root growth due to tube installation were taken, the root parameters are expected to have at most negligible deviations in this respect.

The data collected in this study can be used to develop, calibrate, and validate models of the soil–plant continuum across different scales^30^ with regard to different root zone components such as soil processes, including flow processes^31,32^, root development^33^, and biopores^34^ as well as different model compilations such as single-plant and^33^ multi-plant modeling^35^ or soil water content and root water uptake modeling^36,37^. The data include agronomically relevant information for breeding water-efficient cultivars and for field management under various conditions, which can be directly used by, for example, agronomists and biologists. Furthermore, the root image data provided here can be used to train and benchmark neural networks, since deep learning-based technologies are a fast and continuously developing branch of plant and agronomic data analysis. The images presented in this paper, which correspond to the root data, are – to the best of our knowledge – the largest available MR image collection, covering several years, cultivars, and agronomic treatments. In this context, the advantage of this image collection is twofold. Firstly, we provide more than 160,000 MR images in one freely available and categorized data set. Secondly, we simultaneously publish reference data that can be used for validation. On the one hand, this will help machine learning scientists to develop models, capturing more heterogeneity. On the other hand, soil and plant scientists will benefit directly from the analyzed data. The data set was acquired for the years 2016, 2017, 2018, 2020, and 2021, and will be continued in the future. The data set will thus be added to each year. Data for the years 2012–2015 are partly available, but are not included in this publication. The related above-ground data, including measurements on crop development, transpiration fluxes, and assimilation rates, will be published in a corresponding paper.

## Methods

### Minirhizotron facilities

The data for this publication were acquired at two MR facilities, allowing us to observe root growth through the rhizotubes and to measure 4D geophysical data. A detailed description of the construction of the MR facilities is provided in Cai *et al*.^28^. Here, we provide a basic overview of the facilities and the data acquisition.

**Fig. 1:**
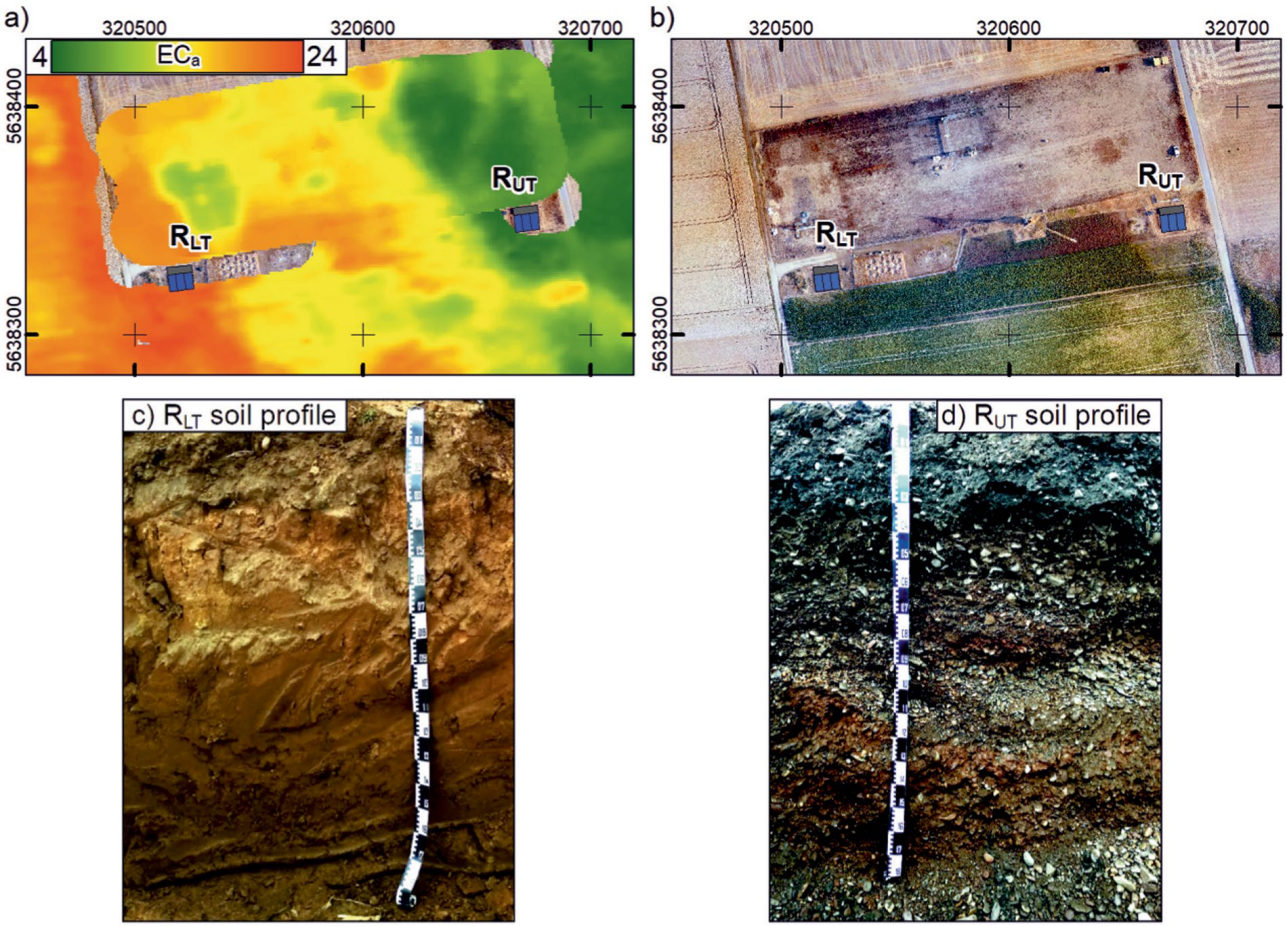
Overview of the location of the minirhizotron(MR)-facilities (**a**) Map of the apparent electrical conductivity (ECa in [mS/m]) measured with the electromagnetic induction (EMI) (vertical diapoles, 9.7 cm depth of investigation, 135 cm coil distance) of the Selhausen test site. Provided by Brogi *et al*.^42^. (**b**) Aerial photograph of the Selhausen test site and the MR-facilities. Both maps are given in WGS 1984 UTM Zone 32 N [m]. For (**a**) and (**b**) the location of the MR-facilities is given by the blues rectangles, the upper terrace facility (R_ut_) and the lower terrace facility (R_lt_), the location of the access trench is indicated with a grey rectangle. (**c,d**) Photos of the soil profiles of the loamy soil at the R_lt_ (**c**) and of stony soil at the R_ut_ (**d**).

The MR facilities are situated within the TERENO (TERrestrial ENvironmental Observatories) Eifel/Lower Rhine observatory near Selhausen, Germany (50°52′N, 6°27′E) (see Fig. 1a). The Selhausen test site was mentioned in various studies ranging from geophysical observations and soil physics to root and plant modeling^36,38–42^. The weather station (SE_BDK_002) is located within the Selhausen test site. The recorded parameters are used to calculate the evapotranspiration with a temporal resolution of 10 min. The data are available in the TERENO Data Discovery Portal (https://ddp.tereno.net/ddp/). The soil at the two MR facilities was deposited by fluvio-glacial sediments of the river Rur catchment during the Pleistocene^28,41,43^. Different river sediments were deposited at each MR facility. The upper terrace sediments consist of gravely, partly stony, and silty sand, and it is here where the upper terrace MR facility (R_ut_) is located. It is classified as Orthic Luvisol with a high stone content (>50%) (Yu *et al*.^27^) according to the World Reference Base for Soil Resources (IUSS Working Group WRB, 2007). The soil at the lower terrace is classified as Cutanic Luvisol (Ruptic, Siltic) (Bauer *et al*.^39^), and it is here where the lower terrace MR facility (R_lt_) is located. The soil organic content and total soil nitrogen (derived from 2020) were 1.14% and 0.116% (0–0.3 m), 0.66% and 0.081% (0.3–0.6 m), and 0.42% and 0.059% (0.6–1 m) in R_lt_ as well as 1.39% and 0.128% (0–0.3 m, with a stone weight of 45%) in R_ut_. The sand, silt, and clay contents are on average 16%, 63%, and 21% (0–1 m, R_ut_) and 32%, 53%, and 15% (0–0.3 m, R_ut_). The different soils cause a 4° morphology incline from R_ut_ towards R_lt_ (see Cai, *et al*.^28^. Due to regular tilling and plowing, a 0.3-m-thick plow layer (Ap horizon) was present in the upper 0.3 m of the two MR facilities (see Fig. 1b,c).

**Fig. 2:**
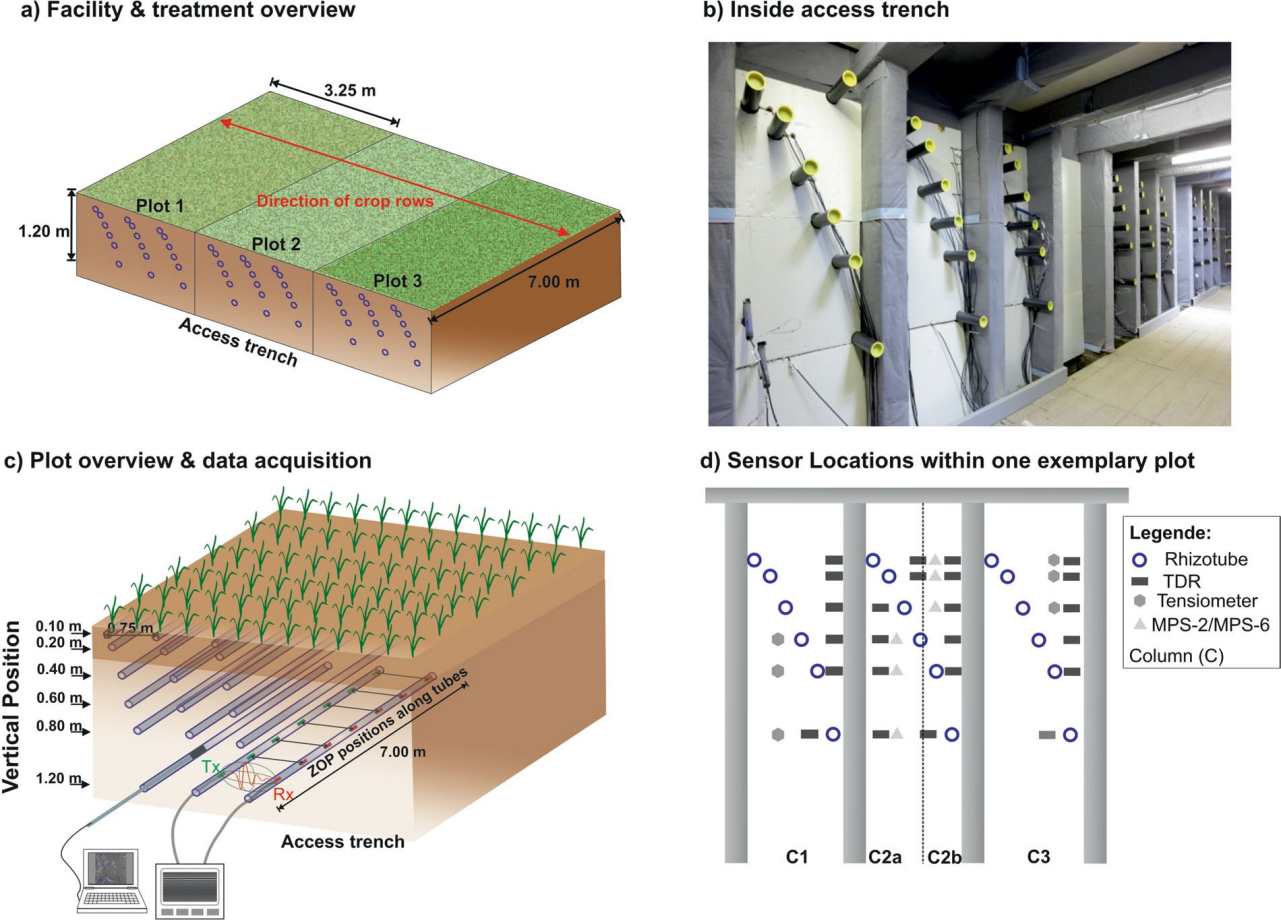
Overview of the Minirhizotron (MR)-facilities. (**a**) Schematic setup of the MR-facilities indicating that at each of the plots a different agricultural treatment was applied for the different growing seasons. The direction of the crop rows is perpendicular to the direction of the rhizotrubes (red arrow). The measurements are carried out from the access trench. (**b**) View within the access trench. (**c**) Overview of one exemplary plot within the MR-facilities with the horizontal crosshole GPR ZOP measurement set up. Transmitter and receiver antennae are labeled Tx and Rx, respectively. Root image measurement are acquired using camera system attached to an index handle. (**d**) Sensor location for one exemplary plot.

To compare different agronomic treatments under the same soil and atmospheric conditions, the two MR facilities were divided into three plots (Fig. 2a). Within the individual plots, three horizontal rhizotubes were installed at each of six different depths between 0.1 m and 1.2 m, each with a length of 7 m. The rhizotubes were embedded at a distance of 0.75 m in the horizontal axis (Fig. 2a). For each crop growing season, a crop row orientation perpendicular to the rhizotubes was chosen. To perform the measurements within the rhizotubes an access trench was built within the ground in front of the plots, from which the rhizotubes can be reached. At R_ut_, the soil was excavated and refilled while installing the rhizotubes, which was due to the high stone content. A plastic foil was installed down to 1.3 m depth to separate the plots. At R_lt_, the soil is undisturbed since the installation was performed by drilling. The soil was precisely compacted layer by layer to the same bulk density as the undisturbed soil (see Cai, *et al*.^28^. For R_ut_, the differences in excess length is negligible, as they are less than <0.02 m. In contrast, for R_lt_, excess lengths are up to 0.10 m. This was taken into account during the processing of the data. Due to soil erosion and soil compaction after tillage and seedbed preparation, the depths of the rhizotubes vary between the individual measurement seasons. The individual rhizotube depths are provided in the repository “Additional_Information”^44^.

In addition to the measurements (GPR and root images) that can be performed within the rhizotubes, various soil sensors are embedded within the soil (see Soil Sensor Data section). Above ground at R_lt_, there is a monitoring system for spectral electrical impedance tomography (sEIT)^45^.

A water reservoir is installed to provide rainwater for irrigation.

### Study design

The MR facilities allow an *in situ* investigation of the soil–plant continuum. To observe the impact of drought stress and planting density on different crops and the impact of crop mixtures on root development, various agronomic treatments were carried out for the different plots. This includes, depending on the growing season, surface water treatment (sheltered, natural/rainfed & irrigated), planting density, sowing date, and different crop cultivar mixtures. In this study, we present the data of multiple crop growing seasons between the years 2016 and 2021. An overview of the individual crop growing seasons and the agricultural treatments is provided in the repository “Additional_Information”^44^.

During the 2016 crop growing season, the goal was to compare different drought stress levels for winter wheat (*Triticum aestivum*, cv. Ambello). A shelter was therefore installed on Plot 1 for both MR facilities. The shelter had a cover, which was removed when no precipitation was forecasted. Plot 2 was left under natural conditions and is also referred to as the rainfed plot. For Plot 3, irrigation pipes were installed and the soil was irrigated regularly. The individual irrigation values can be found in the “Additional_Information”^44^. For crop growing seasons 2017 & 2018, *Zea mays* (cv. Zoey) was chosen and the shelter needed to be removed due to the height of the crop. This resulted in two rainfed plots (Plot 1 and Plot 2). As before, Plot 3 was irrigated. In 2018, the influence of the sowing date and the planting density was investigated on Plot 1 for R_ut_ and R_lt_, respectively.

**Fig. 3:**
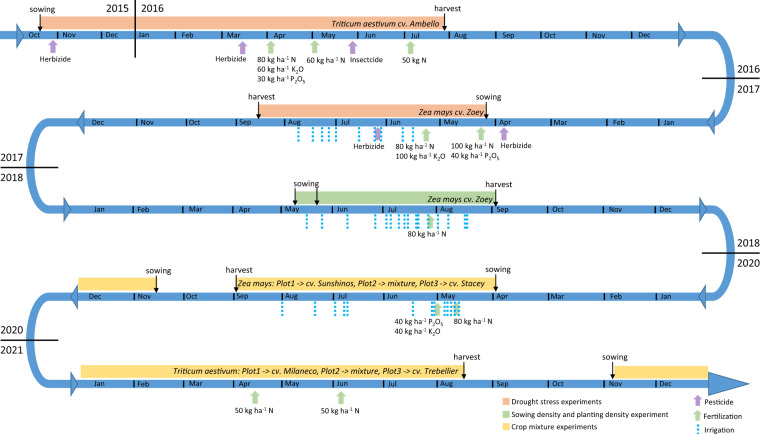
Overview of the experimental timeline including cultivars and management actions, such as sowing, harvest, pesticide applications and irrigation.

Since the 2020 crop growing season, the focus of research was on comparing the different crop root architectures of cultivars – purely sown and in a cultivar mixture with alternating rows. To explore the beneficial effects of mixing deep and shallow rooting cultivars, one cultivar chosen was always a deep rooting, while the other one was a shallow rooting cultivar. The surface water treatment was therefore uniform for all three plots. Irrigation was only applied to all crops under heavy drought conditions when the crops showed severe drought stress symptoms. For the 2020 crop growing season, two different *Zea mays* cultivars (cv. Sunshinos and cv. Stacey) were sown on Plot 1 and Plot 3, respectively. The cultivar mixture was sown on Plot 2. For the 2021 growing season, winter wheat (*Triticum aestivum*) with two different cultivars (cv. Milaneco and cv. Trebelir) was again sown on Plot 1 and Plot 3, respectively. The mixture was sown on Plot 2. In 2021, irrigation was not required since the winter wheat was sufficiently supplied by precipitation and the crops did not show any stress symptoms (Fig. 3). In order to perform destructive measurements above and below ground in 2020 and 2021, a replication field (extra field (EF)) next to R_lt_ was sown. The EF had the same dimension and plot design as the MR facilities and was located on the west side of the facility (see Above-Ground Data section).

### Ground-penetrating radar data

#### Crosshole ground-penetrating radar data acquisition at the minirhizotron facilities

The time-lapse GPR data were collected using a 200 MHz PulseEKKO borehole system manufactured by Sensors and Software (Canada). Crosshole zero-offset-profiling (ZOP) measurements were carried out, with the transmitter (Tx) and receiver antennae (Rx) located within neighboring rhizotubes. Both antennae were simultaneously pulled in parallel positions along the length of the rhizotubes, with a spacing of 0.05 m between the individual ZOP positions. An electromagnetic (EM) wave is emitted by Tx, which is sent through the soil and then recorded by Rx. Changes in soil and root properties between the rhizotubes affect the measured GPR traces and, therefore, information about the medium parameters can be obtained (more information can be found in Klotzsche *et al*.^26^. Due to the different rhizotube lengths of both MR facilities, the length over which the ZOPs are collected is 6.70 m and 6.40 m, resulting in 115 and 109 traces for R_ut_ and R_lt_, respectively.

For a time-zero calibration, wide-angle reflection and refraction (WARR) measurements are carried out within the access trench. Here, Rx antennae are moved over a distance of 6.0 m with a step size of 0.1 m, while the Tx antennae are fixed at the zero location. At least four calibration measurements per MR facility and measurement day were performed to capture daily variations of the time-zero (see GPR Data Processing section).

In contrast to the root images, which capture the soil in contact with the rhizotubes, the ZOP measurements investigate the soil between two rhizotubes. A 1D horizontal permittivity profile is thus obtained. For the measurements seasons 2016–2018, only one horizontal permittivity plane was measured per depth. For Plot 1 and Plot 2, this were the slices between column C1 and C2, and for Plot 3 between column C2 and column C3. In 2020, two main planes were measured per depth; occasionally only one plane was measured with the same configuration as for the previous measurement seasons. Table 1 indicates that the number of horizontal permittivity planes was measured per measurement date.

**Table 1:** Detailed overview of the GPR data acquired during growing season 2016, 2017, 2018, 2020 and 2021.

		2016		2017		2018		2020		2021	
no	fac	date	pl	date	pl	date	pl	date	pl	date	pl
1	R_ut_	03.02.2016	12	26.04.2017	15	25.04.2018	15	19.03.2020	12	—	—
	R_lt_	03.02.2016	—	26.04.2017	14	25.04.2018	14	—	—	25.11.2020	29
2	R_ut_	30.03.2016	15	03.05.2017	15	02.05.2018	15	12.05.2020	30	—	—
	R_lt_	30.03.2016	10	03.05.2017	14	02.05.2018	14	—	—	02.12.2020	30
3	R_ut_	08.04.2016	15	10.05.2017	14	09.05.2018	15	28.05.2020	30	—	—
	R_lt_	08.04.2016	15	10.05.2017	14	09.05.2018	14	—	—	14.12.2020	29
4	R_ut_	14.04.2016	15	17.05.2017	15	14.05.2018	15	03.06.2020	30	—	—
	R_lt_	14.04.2016	15	17.05.2017	14	14.05.2018	14	—	—	14.01.2021	29
5	R_ut_	20.04.2016	15	23.05.2017	15	24.05.2018	15	10.06.2020	30	—	—
	R_lt_	20.04.2016	15	23.05.2017	11	24.05.2018	14	—	—	27.01.2021	29
6	R_ut_	28.04.2016	15	31.05.2017	15	20.06.2018	15	17.06.2020	25	—	—
	R_lt_	28.04.2016	15	31.05.2017	14	20.06.2018	14	—	—	10.02.2021	29
7	R_ut_	04.05.2016	15	07.06.2017	15	27.06.2018	15	06.07.2020	29	04.03.2021	30
	R_lt_	04.05.2016	15	07.06.2017	14	27.06.2018	14	—	—	—	—
8	R_ut_	12.05.2016	15	14.06.2017	15	04.07.2018	15	15.07.2020	30	—	—
	R_lt_	12.05.2016	15	14.06.2017	14	04.07.2018	14	—	—	09.03.2021	—
9	R_ut_	19.05.2016	15	21.06.2017	15	09.07.2018	15	23.07.2020	5	11.03.2021	30
	R_lt_	19.05.2016	15	21.06.2017	14	—	14	—	—	11.03.2021	—
10	R_ut_	25.05.2016	15	05.07.2017	15	11.07.2018	15	27.07.2020	30	19.03.2021	24
	R_lt_	25.05.2016	15	05.07.2017	14	11.07.2018	14	—	—	19.03.2021	—
11	R_ut_	02.06.2016	15	12.07.2017	15	18.07.2018	15	05.08.2020	5	30.03.2021	15
	R_lt_	02.06.2016	14	12.07.2017	14	18.07.2018	14	—	—	30.03.2021	29
12	R_ut_	09.06.2016	15	19.07.2017	15	19.07.2018	15	—	—	15.04.2021	30
	R_lt_	09.06.2016	15	19.07.2017	14	19.07.2018	14	—	—	15.04.2021	—
13	R_ut_	13.06.2016	15	27.07.2017	15	20.07.2018	15	—	—	14.07.2021	30
	R_lt_	13.06.2016	15	27.07.2017	14	20.07.2018	14	—	—	22.07.2021	—
14	R_ut_	20.06.2016	15	02.08.2017	15	25.07.2018	15	—	—	28.07.2021	30
	R_lt_	20.06.2016	14	02.08.2017	14	25.07.2018	14	—	—	28.07.2021	29
15	R_ut_	27.06.2016	15	09.08.2017	15	01.08.2018	15	—	—	04.08.2021	30
	R_lt_	27.06.2016	14	09.08.2017	14	01.08.2018	14	—	—	04.08.2021	28
16	R_ut_	04.07.2016	15	14.08.2017	15	08.08.2018	15	—	—	18.08.2021	15
	R_lt_	27.06.2016	15	09.08.2017	15	01.08.2018	15	—	—	04.08.2021	30
17	R_ut_	20.07.2016	15	23.08.2017	15	15.08.2018	15	—	—	—	—
	R_lt_	20.07.2016	15	23.08.2017	14	15.08.2018	14	—	—	25.08.2021	30
18	R_ut_	27.07.2016	15	30.08.2017	15	22.08.2018	15	—	—	—	—
	R_lt_	27.07.2016	15	30.08.2017	14	22.08.2018	14	—	—	31.08.2021	23
19	R_ut_	01.08.2016	15	06.09.2017	15	05.09.2018	15	—	—	10.09.2021	30
	R_lt_	01.08.2016	15	06.09.2017	14	05.09.2018	14	—	—	10.09.2021	19
20	R_ut_	08.08.2016	15	13.09.2017	15	17.09.2018	15	—	—	29.09.2021	30
	R_lt_	08.08.2016	15	13.09.2017	14	17.09.2018	14	—	—	—	—
21	R_ut_	15.08.2016	15	20.09.2017	15	24.09.2018	15	—	—	03.11.2021	30
	R_lt_	15.08.2016	15	20.09.2017	14	24.09.2018	14	—	—	03.11.2021	27
22	R_ut_	—	—	27.09.2017	15	02.10.2018	15	—	—	—	—
	R_lt_	—	—	27.09.2017	14	02.10.2018	14	—	—	—	—

#### Ground-penetrating radar data processing

**Fig. 4:**
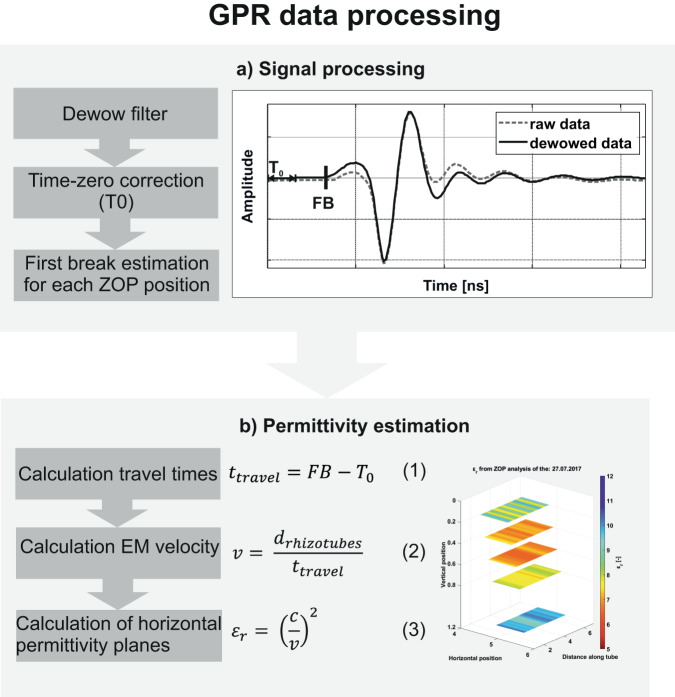
GPR processing steps.

From horizontal GPR crosshole ZOP measurements, we can derive the relative dielectric permittivity *ε*_*r*_, which can be transformed into SWC using appropriate petrophysical relationships. All the required pre-processing steps are explained in detail by Klotzsche *et al*.^26^. Here, we highlight the most important aspects. Firstly, a dewow filter is applied, which reduces low-frequency noises on the GPR data. Secondly, a time-zero (T0) correction of the ZOP data is performed and thirdly, the first breaks (FB) of the signals are estimated (Fig. 4a).

Following this processing procedure, the EM wave travel times between the neighboring rhizotubes for each ZOP position are obtained. Since the horizontal spacing between the neighboring rhizotubes (d_r__hizotubes_) is known to be 0.75 m, the EM wave velocity *v* for each ZOP position can be calculated using the obtained travel times (t_travel_), see Fig. 4b. As suggested by Jol^46^, when considering low-loss and non-magnetic soils the EM velocity *v* can be transformed into the relative dielectric permittivity *ε*_*r*_ of the bulk material with

1 $$v=\frac{c}{\sqrt{{\varepsilon }_{r}}}$$

where *c* is the speed of light (0.3 m/ns).

Because of the presence of the soil sensors and pertaining cables in the first 0.75 m away from the facility wall, GPR measurements were made between 1 and 7 m away from the facility wall. Close to the surface (depth of 0.1 m) the radar wave interferences of the critically refracted air wave and the direct wave^26^ occur. Therefore, these data were excluded. Additionally, at R_lt_, an sEIT system is installed and the metal parts interfere with the GPR waves. Therefore, at a depth of 0.2 m, where the sEIT system is located, the data were also excluded.

GPR-derived permittivity can be transformed into the soil water content (SWC), which provides a parameter that is directly used in soil science. This is achieved by using different conversion formulas, which are based on empirical relationships and petrophysical, volumetric mixing models (see Huisman *et al*.^47^ and Steelman *et al*.^48^). In this data descriptor, we provide the permittivity values to ensure that the conversion can be chosen by the user of the data. In the past, we have used two conversions, the Topp’s equation^49^ and the complex refractive index model (CRIM)^48^ (see Klotzsche, *et al*.^26^ and the Dielectric Permittivity to Soil Water Content section).

### Root images

#### Root image acquisition at the minirhizotron facilities

Images of roots and the surrounding soil were captured through the transparent rhizotubes. The amount of images obtained varied depending on the vegetation and the progress of root development. To save resources, the depth of measurement was continuously increased at the beginning of each growing season as root depth increased. Meticulous care was taken not to omit any root depth at which roots were already present. A measurement produces always 40 images per tube. Half of the images were taken 80° clockwise and the other half were taken 80° counter-clockwise from the top point of the rhizotubes. Two different camera systems were used over time to take the images. The camera used in 2016, and for most measurements in 2017, was manufactured by Bartz (Bartz Technology Corporation). The camera used for some of the images taken in 2017 and for all images taken in 2018, 2020, and 2021 was produced by VSI (Vienna Scientific Instruments GmbH).The photographed area differs depending on the camera (Table 2). Table 3 provides a detailed overview of the images taken over the different growing seasons.

**Table 2:** Overview of the camera-systems and experiment timeline of minirhizotron images acquisition.

camera system	Bartz	VSI
resolution (px)	1508 × 1020	2060 × 2060
real size (mm)	16.5 × 23.5	20 × 20
wavelength (nm)	400–780	400–780
growing season	2016 & 2017	2017 & 2018 & 2020 & 2021

**Table 3:** Detailed overview of the images taken at the growing season 2016, 2017, 2018, 2020 and 2021.

		2015/16		2017		2018		2020		2020/21	
no	fac	date	img	date	img	date	img	date	img	date	img
1	R_ut_	16.11.2015	719	08.06.2017	480	23.05.2018	440	02.07.2020	1,160	24.02.2021	1,480
	R_lt_	16.11.2015	720	08.06.2017	584	23.05.2018	720	13.08.2020	1,760	14.01.2021	600
2	R_ut_	26.11.2015	1,070	29.06.2017	1,800	30.05.2018	480	13.08.2020	1,800	03.03.2021	1,440
	R_lt_	26.11.2015	1,073	22.06.2017	1,800	30.05.2018	720	—	—	27.01.2021	920
3	R_ut_	17.12.2015	1,799	06.07.2017	1,800	07.06.2018	960	—	—	11.03.2021	1800
	R_lt_	17.12.2015	1,439	29.06.2017	2,160	07.06.2018	1,075	—	—	04.02.2021	1,280
4	R_ut_	02.02.2016	1,518	13.07.2017	1,800	18.06.2018	1,280	—	—	01.04.2021	440
	R_lt_	21.01.2016	1,795	06.07.2017	2,160	18.06.2018	1,436	—	—	24.02.2021	1,320
5	R_ut_	12.02.2016	1,789	20.07.2017	1,800	26.06.2018	1,400	—	—	08.04.2021	2,160
	R_lt_	12.02.2016	1,798	13.07.2017	2,160	26.06.2018	1,800	—	—	03.03.2021	1,280
6	R_ut_	26.02.2016	1,795	27.07.2017	1,200	05.07.2018	1,638	—	—	22.04.2021	1,560
	R_lt_	26.02.2016	2,155	20.07.2017	2,160	18.07.2018	2,156	—	—	10.03.2021	1,640
7	R_ut_	14.03.2016	1,792	02.08.2017	1,840	18.07.2020	1,760	—	—	21.05.2021	2,160
	R_lt_	14.03.2016	2,158	27.07.2017	1,430	01.08.2018	2,159	—	—	07.04.2021	2,000
8	R_ut_	26.03.2016	1,837	10.08.2017	1,959	01.08.2018	1,680	—	—	01.06.2021	520
	R_lt_	24.03.2016	2,155	02.08.2017	2,157	23.08.2018	2,159	—	—	21.05.2021	1,960
9	R_ut_	07.04.2016	2,157	23.08.2017	2,120	16.08.2018	1,676	—	—	07.06.2021	240
	R_lt_	07.04.2016	2,158	10.08.2017	2,154	—	—	—	—	01.06.2021	1,960
10	R_ut_	13.04.2016	2,160	12.09.2017	1,800	—	—	—	—	—	—
	R_lt_	13.04.2016	2,157	24.08.2017	2,159	—	—	—	—	—	—
11	R_ut_	29.04.2016	2,154	—	—	—	—	—	—	—	—
	Rlt	29.04.2016	2,157	12.09.2017	2,150	—	—	—	—	—	—
12	R_ut_	06.05.2016	2,154	—	—	—	—	—	—	—	—
	R_lt_	06.05.2016	2,144	—	—	—	—	—	—	—	—
13	R_ut_	13.05.2016	2,151	—	—	—	—	—	—	—	—
	R_lt_	13.05.2016	2,155	—	—	—	—	—	—	—	—
14	R_ut_	20.05.2016	2,156	—	—	—	—	—	—	—	—
	R_lt_	20.05.2016	2,155	—	—	—	—	—	—	—	—
15	R_ut_	27.05.2016	2,152	—	—	—	—	—	—	—	—
	R_lt_	27.05.2016	2,153	—	—	—	—	—	—	—	—
16	R_ut_	03.06.2016	2,108	—	—	—	—	—	—	—	—
	R_lt_	03.06.2016	2,153	—	—	—	—	—	—	—	—
17	R_ut_	09.06.2016	2,114	—	—	—	—	—	—	—	—
	R_lt_	09.06.2016	2,083	—	—	—	—	—	—	—	—
18	R_ut_	16.06.2016	2,111	—	—	—	—	—	—	—	—
	R_lt_	16.06.2016	2,142	—	—	—	—	—	—	—	—
19	R_ut_	23.06.2016	2,087	—	—	—	—	—	—	—	—
	R_lt_	23.06.2016	2,006	—	—	—	—	—	—	—	—

#### Root image data processing

The post processing of the images was performed by an automated analysis pipeline including neural network segmentation and automated feature extraction following the analysis pipeline of Bauer *et al*.^50^. Neural network training and image segmentation were performed with the “RootPainter”^51^ software. Firstly, the roots were segmented by a CNN. As part of the process, the roots are separated from the background and extracted as binary image data. A small subset of the root images is used as training data to train the CNN. The evaluation of the models was performed with the F1-score (>0.7 for each model used). More information on the models can be found in Bauer *et al*.^50^. The resulting neural network model was then used for the segmentation of the roots. The segmentation of the images was performed in a batch process. Secondly, the morphological features were extracted by the automated feature extraction program “RhizoVision Explorer”^52^. This includes multiple automated steps for thresholding obstacles and filling holes smaller than 0.2 mm as well as the skeletonization of the roots and the feature derivation from the skeletonized roots.

**Fig. 5:**
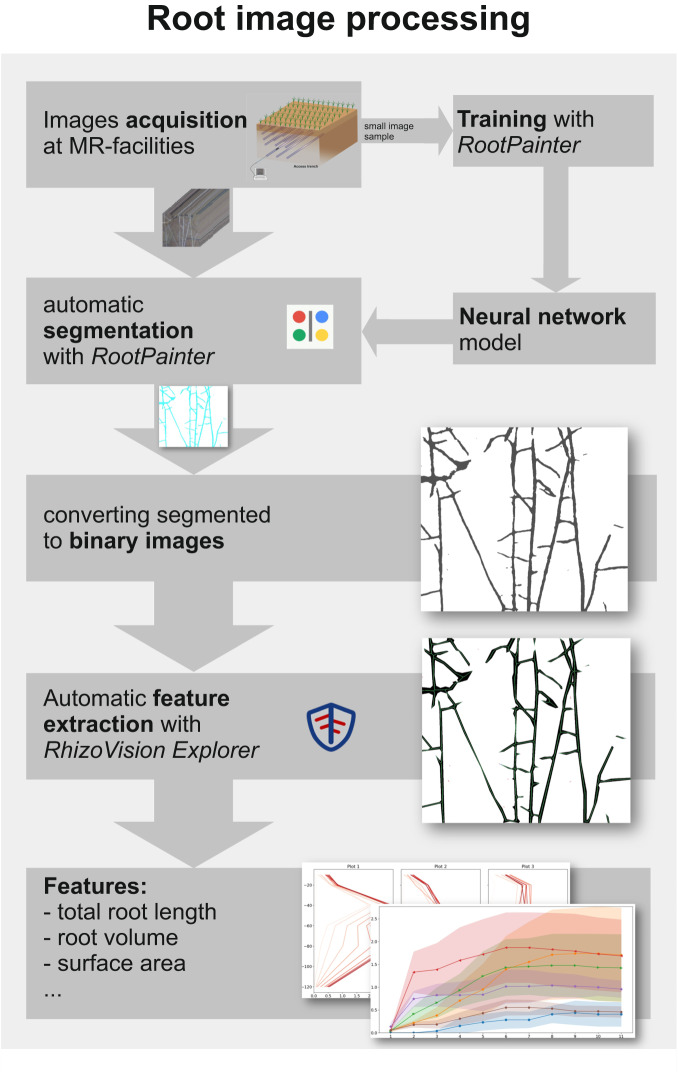
Root image processing steps.

The root system parameters provided by the automated analysis include the total root length, branch points, branching frequency, diameter (average, maximum, median), network area, perimeter, amount of root tips, volume, and surface area^50^ (Fig. 5).

### Soil coring in the extra field

Soil coring was performed in the EF (extra field established next to R_lt_) dedicated to destructive belowground measurements in 2020 (maize) and 2021 (winter wheat). The soil next to R_ut_ is not homogeneous, which is why a representative replica was not feasible. The maize roots were extracted once on July 14, 2020 when the crops were in BBCH 65, whereas the winter wheat roots were extracted on June 16, 2021 when the crops were in BBCH 69. The soil was cored using a root auger with an inner diameter of 0.9 m and a length of 1.0 m, and the cores were drilled directly around the plant. The soil core was then divided into 0.1 m pieces and filled into plastic bags. For maize in 2020, four replicates were taken in Plot 1 and four replicates in Plot 3 of the EF (no core was taken in the cultivar mixture treatment – Plot 2). For winter wheat in 2021, one replicate was taken in Plot 1, one in Plot 3, and two in Plot 2 of the EF (one core for each variety in the cultivar mixture). The soil samples were then put into refrigerators and processed step by step. The samples were later soaked in tap water, washed, and passed through several sieves with mesh sizes of 1.00 mm, 0.83 mm, and 0.5 mm until the coarsest soil and residues were cleared. The roots were subsequently stored in tap water at 3 °C until they were scanned with an EPSON scanner (HP Expression 1100XL). The roots of each sample were laid (preferably without overlaps) into an acrylic glass plate filled with tap water and were subsequently scanned. The images of the scanned roots were processed using a similar procedure as for the minirhizotron images, resulting in the total length estimation of the roots and the root length density^53^.

### Soil sensor data

All plots within the two MR facilities have the same layout. Each plot contains three horizontal rhizotubes per depth but the soil sensors are distributed into four columns, with the middle section divided into two columns, column C2a and C2b (see Fig. 2c). For each column, there are four TDR-sensors installed for each of the six depths. For the tensiometers and the soil water potential and soil temperature sensors, one sensor is installed for each depth. The distribution over the four columns is shown in Fig. 2c.

To measure the soil water potential for dry soil conditions and to acquire the soil temperature, MPS-2 sensors manufactured by Decagon Devices, Inc., US are used. The soil water potential is measured in a range of −9 kPa to −100,000 kPa (pF 1.96 to pF 6.01) with a resolution of 0.1 kPa. The accuracy is of ±(25% of reading + 2 kPa) over the range of −9 to −100 kPa and proven to be higher for drier conditions until permanent wilting point (−1,500 kPa) under lab conditions and −4,500 kPa under field conditions by the manufacturer. The soil temperature is measured in a range of −40 °C to 60 °C with a resolution of 0.1 °C. The soil water potential for wet soil conditions is measured using T4 pressure transducer tensiometers manufactured by UMS GmbH, Germany. The measurement range is −85 kPa to + 100 kPa with an accuracy of ± 0.5 kPa. To acquire and record the soil sensor data, all sensors – with the exception of the TDR sensors – are connected to a DataTaker DT85 manufactured by Omni Instruments Ltd, UK. The TDR sensors were manufactured by the institute’s technicians and consist of three rods, with a length of 200 mm and a spacing of 26 mm. The TDR sensors are connected to institute-made multiplexers (50C81-SDM), providing a lower relative error (>1%) then commercial system. To acquire and record the data, the multiplexers are connected to a TDR100 Time-Domain Reflectometer manufactured by Campbell Scientific, Inc., US. Because of the high stone content at R_lt_ the relationship of SWC and dielectric permittivity measured by the TDR was calibrated in the lab^28^. For information on SWC calculation see Dielectric Permittivity to Soil Water Content section.

### Soil water content using a mobile frequency domain reflectometry device

In addition to the soil sensors (see Soil Sensor Data section), the soil water content was measured using the mobile FDR device that employs the HH2 moisture sensor with the ThetaProbe ML3 (ecoTech Umwelt-Messsysteme GmbH, Bonn, Germany). Due to the nature of the soil at R_ut_, the soil moisture was only measured for the topsoil, while for the R_lt_ and EF, the soil water was measured at depths of 0 m, 0.30 m, 0.6 m, and 0.9 m. In total, the soil water was measured ten times in each plot of the R_ut_, six times in each plot of the R_lt_, and eleven times in each plot of the EF over the crop growing season. The sensor was always placed between crop rows.

### Soil sampling

In September 2020, a new irrigation tank was installed at R_lt_ and undisturbed soil samples were taken from the trench for the new tank. The samples were taken from several depths and analyzed in the in-house soil physics lab. The soil hydraulic parameters were measured using the HYPROP (Meter, München, Germany) method^54^ and a WP4 Dewpoint Potentiometer (Decagon Devices, WA, USA). The saturated hydraulic conductivity was derived using the KSAT system (Meter, München, Germany). Soil texture was determined according to DIN ISO 11277 using the pipette method combined with wet sieving^55^.

The soil hydraulic properties can be found in the “Additional_Information”^44^.

## Data Records

All data were uploaded to Geonetwork in accordance with ISO 19115. The data were persistently stored and will be regularly updated (see Usage Notes). The data were subdivided according to the characteristics of the sensing method and data type. GPR data^56^, root data^57^ root images^58^, and soil sensor data^59^ are each available with a DOI, providing a link to a repository. Within these repositories, the data were subdivided by year of measurement. In the GPR data^56^ repository, one folder for each year contains two CSV files – one for all measurements performed on each facility in the corresponding year. The root image data repository contains a CSV file for each root trait measured in the corresponding year and facility.

**Fig. 6:**
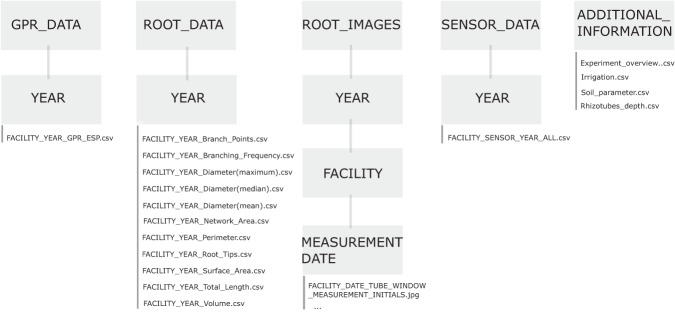
Folder structure of the repositories.

The root images^58^ were organized by year and facility. For each measurement date, one folder (labeled: YYYYMMDD) contains all images measured on that date in the corresponding facility. The sensor data^59^ repository contains one file for each sensor type and facility, corresponding to the year the data were obtained. The file names are explained in Table 4 and the repository structures in Fig. 6. The data can be downloaded using the following links:

**Table 4:** Overview of the repository content and data labelling.

repository	data label	size
GPR_Data	*FACILITY*_ *YYYY*_GPR_EPS.csv	2.68 *MB*
Root_Data	*FACILITY*_*YYYY*_*ROOT PARAMETER*.csv	21.6 *MB*
Root_Images	*FACILITY YYYYMMDD*_*TUBE*_*WINDOW*_*MEASUREMENT*_*INITIALS*.jpg	199 *GB*
Soil sensors_Data	*FACILITY*_ *SENSOR YYYY*_ALL.csv	103 *MB*
Additional_Information	experiment, irrigation and soil overview (CSV)	1 *MB*

The labels always contain the facility name (R_ut _or R_lt_) and the year the data haven been obtained. For the root images, each image is also labeled according to exact date (year (YYYY), month (MM), day (DD)), tube and position it was taken.

GPR data^56^: 10.34731/cg3t-nb88,

Root data^57^: 10.34731/7x05-2r96,

Root images^58^: 10.34731/5zwe-t974,

Soil sensor data^59^: 10.34731/ffsk-sy65,

Additional Information^44^: 10.34731/st8e-4082.

Some root image data have been previously used and published. Root length data from 2016 were used by Nguyen *et al*.^60^. Root length data obtained from the images and the soil moisture values, measured by TDR and MPS-2 sensors on both facilities in 2016 and 2017 were used by Morandage *et al*.^29^. The root image data of R_ut_ from June 8, July 13, and September 12, 2017 were used by Nguyen *et al*.^61^. However, the root lengths used in these three studies were obtained by a different method and are based on a manual single root annotation^62^. The root length data of R_ut_ and R_lt_ from 2017 were published by Bauer *et al*.^50^ to validate the analysis pipeline used to extract all root data. The GPR data and the mean soil water content values calculated from TDR sensors from 2016 and 2017 have already been partly used by Klotzsche *et al*.^26^.

## Technical Validation

### Ground-penetrating radar data

The GPR permittivities^56^ were manually checked for plausibility and unreliable data were excluded. Implausible permittivity outliers were manually detected and removed.

### Root images

The root data^57^ derived from the minirhizotron images^58^ were automatically analyzed by the pipeline following Bauer *et al*.^50^ using deep neural networks and automated feature extraction^51,52^. Using this approach, part of the total root length data has been representatively compared to a manual annotation of the images. Approximately 36,500 images were used for validation. The correlation of total root length values obtained from the same images by manual annotation and automated analysis is very high (*r* = 0.9)^50^.

### Soil sensor data

The data^59^ of the different sensor types were filtered for the different measurement ranges listed in the Methods Soil Sensor Data section. To remove outliers, we applied a Hampel filter, which involves a sliding window being moved over the data. As a window size, we used 10 data points for each size of the element, which corresponds to 5 h for the tensiometers and MPS-2 to 10 h for the TDR sensors. For the element, we calculated the median and the standard deviation. If the element deviated more than one time the standard deviation, then the element is replaced by the median^63^. Additionally, the data from the different soil sensors were manually checked for plausibility and unreliable data were excluded. The TDR sensor data were filtered for errors in the TDR wave recordings and data for different dates and sensors were excluded.

## Usage Notes

**Fig. 7:**
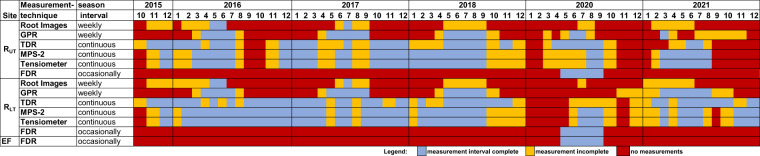
Data availability for the measurement seasons 2016–2021.

Figure 7 provides information on which periods of data are available for the different measurement seasons and the different measurement techniques. In 2019, no crops were sown on the MR facilities due to a project change. In 2020 and 2021, the data sets do not cover the whole growing period due to technical issues within the access trench and the measurement systems. Different measurement intervals were used for the different measurement techniques. For the root images and the GPR measurements, weekly measurements were performed when possible during the vegetation period. The interval was adjusted to a biweekly period for the root images when the root growth stagnated. The availability of the sensor data (TDR, Tensiometer & MPS-2) depends on the technical state of the measuring devices, and in 2020 and 2021 there were problems with the data recording system. The measurements should be recorded as continuous measurements with measuring intervals of 30 min for tensiometers and MPS-2 sensors and 1 hour for TDR sensors. All timestamps are UTC + 1.

### Soil sensor data

Due to the measurement interval and the sensitivity of the TDR permittivity time series results, we suggest applying a median filter or similar filters to the TDR data set to smooth the data as well as to remove the outliers, as mentioned above.

### Dielectric permittivity to soil water content

Using the geophysical measurement techniques mentioned in this study, we provide the dielectric permittivity of the soil. Point information is provided by the TDR measurements and spatial information along the rhizotubes is provided by the GPR measurements. The dielectric permittivity can be converted to the soil water content. In the past, literature using TDR and GPR data measured within the MR facilities have used the empirical Topp’s equation^49^ and the petrophysical relationships referred to as the complex refractive index model (CRIM) (see^47^). The Topp’s equation is valid for sandy loam to clay and requires the bulk permittivity of the soil (*ε*_*r*_) to derive the soil water content (SWC):

2 $$SWC=-5.3\times 1{0}^{-2}+2.92\times 1{0}^{-2}{\varepsilon }_{r}-5.5\times 1{0}^{-4}{\varepsilon }_{r}^{2}+4.3\times 1{0}^{-6}{\varepsilon }_{r}^{3}.$$

For the petrophysical relationship CRIM, which considers the different dielectric components of the soil (air, soil matrix, and soil water), we obtain

3 $$SWC=\frac{\sqrt{{\varepsilon }_{r}}-(1-\phi )\sqrt{{\varepsilon }_{s}}-\phi }{\sqrt{{\varepsilon }_{w}}-1}.$$

For the CRIM approach, additional parameters such as the porosity *ϕ* and the permittivity of the soil matrix *ε*_*s*_, air (*ε*_*a*_ = 1) and water (*ε*_*w*_ = 84, at 10 °C) are necessary. The permittivity of the soil matrix is 4.7 and 4.0 for R_ut_ and R_lt_, respectively^64^. The porosity in the plow layer is considered to be 0.33 and 0.4 for R_ut_ and R_lt_, respectively. For underlying subsoil, the porosity is considered to be 0.25 and 0.35, respectively^38^. In particular, for R_ut_, we recommend using the CRIM relationship instead of the Topp’s equation due to the high stone content.

### Soil hydraulic parameters

To provide information on, for example, rhizosphere modeling, we provide an overview of the soil hydraulic parameters, which were derived for the MR facilities using different methods. In Cai *et al*.^36^, soil hydraulic parameters (SHP) for both MR facilities can be estimated. These were derived by inverse modeling using soil water content, potential measurements, and root observations of winter wheat. Yu *et al*.^27^ and Jadoon *et al*.^40^ estimated the SHP using hydrogeophysical inversion for R_ut_ and R_lt_, respectively. The SHP for R_lt_ was derived by an inverse parameter estimation using a 1-dimensional CO_2_ transport and carbon turnover model, with direct soil sampling and laboratory analysis by Bauer *et al*.^39^.

### Updates

The data corresponding to this paper will be updated regularly on a yearly basis once the analysis is finalized. The updated data can be downloaded from these DOIs:

GPR data: 10.34731/renq-an61,

Root data: 10.34731/jnhr-ke36,

Root images: 10.34731/jgd1-tq27,

Soil sensor data: 10.34731/rb0q-a208,

Additional Information: 10.34731/ke7b-a021.

### Above-ground data

The related above-ground data are managed by the Crop Science group of the Institute of Crop Science and Resource Conservation (INRES), University of Bonn, and will be available upon demand in a future data paper. These data have been partially published in Nguyen *et al*.^60^,^61^,^65^. The data measured within the EF were carried out by the project partner at INRES.

## Data Availability

Custom code was used to process the data. For the GPR Data we used MATLAB version: 9.13. 0 (R2022b) to run the codes. The root image processing and soil sensor data is run with Python 3.10.10. Processing codes for the roots images can be found in the Supporting Material for Bauer *et al*. at 10.34731/pbn7-8g89. The soil water content data measured with the FDR device was processed using R version 4.0.2. The custom codes can not be made publicly accessable due to copyright issues, but are available upon request, by contacting the corresponding or senior author.
